# Accelerating Proficiency in Minimally Invasive Autopsy for Infectious Disease Diagnosis and Research: A Strategic Framework

**DOI:** 10.1590/0037-8682-0037-2026

**Published:** 2026-09-18

**Authors:** Fabiana Santana Souza, Carlos Gustavo Regis-Silva, Ricardo Khouri, Luiz Antônio Rodrigues de Freitas, Milena Botelho Pereira Soares, Bruno Solano de Freitas Souza, Carolina Kymie Vasques Nonaka, Reneé Amorim dos Santos Félix, Maria Fernanda Rios Grassi, Washington Luis Conrado dos-Santos, Geraldo Gileno de Sá Oliveira

**Affiliations:** 1 Fundação Oswaldo Cruz, Instituto Gonçalo Moniz, Salvador, BA, Brazil.; 2 Universidade Federal da Bahia, Faculdade de Medicina, Salvador, BA, Brazil.; 3 Centro Universitário SENAI/CIMATEC, Instituto SENAI de Sistemas Avançados de Saúde (ISI-SAS), Salvador, BA, Brasil.; 4 Hospital São Rafael, Salvador, BA, Brasil.; 5 Escola Baiana de Medicina e Saúde Pública (EBMSP), Salvador, BA, Brasil.

**Keywords:** Ultrasound-guided minimally invasive tissue sampling, Autopsy, Sensitivity and Specificity, Tissue and Organ Harvesting, Contaminants

## Abstract

**Background:**

Conventional autopsy is the gold standard for postmortem examination, but its use is constrained by biosafety, infrastructure, and acceptability concerns. Ultrasound-guided minimally invasive autopsy and ultrasound-guided minimally invasive tissue sampling (US-MITS) offer practical alternatives for tissue sampling in infectious diseases. The aim of this study was to present a standardized protocol for multiorgan US-MITS and evaluate its performance in terms of success rates, accuracy, and sample quality.

**Methods:**

An observational study was conducted on 13 decedents with reverse transcription-polymerase chain reaction (RT-PCR)-confirmed coronavirus disease 2019 in Salvador, Brazil. Using a portable ultrasound machine and 14G semi-automatic Tru-Cut needles, samples were collected from the skin, liver, spleen, kidneys, pancreas, lungs (four-quadrant bilateral sampling), heart, brain, and skeletal muscle. The workflow included routine histological examinations (hematoxylin and eosin, and special stains), electron microscopy, and molecular testing via Ion Torrent sequencing for severe acute respiratory syndrome coronavirus 2 variant calling. Performance was evaluated by measuring the success rates and accuracy (ratio of target tissue to contaminants) using high-resolution slide scanning and digital morphometry.

**Results:**

Sampling success ranged from 77% to 100% across the heart, lungs, liver, spleen, and kidneys. Four-quadrant lung sampling yielded comprehensive histological representation. Contamination was generally low, except in the pancreas and lower medial lung quadrant. Tissue suitable for electron microscopy was obtained from most organs; severe acute respiratory syndrome coronavirus 2 was detected in all 13 lungs, and variants were assigned in 10 cases.

**Conclusion:**

With minimal training, teams can perform US-MITS. Using a traceable, standardized workflow accelerates proficiency while enabling diagnostic-grade samples to be produced for histological examinations, ultrastructural analysis, and molecular testing in resource-limited and outbreak settings.

## INTRODUCTION

Emerging and re-emerging infectious diseases continue to pose major global health challenges, frequently causing epidemics with high morbidity and mortality^1^ ^-^ ^8^. Investigating fatal cases is essential to improve clinical management, elucidate disease mechanisms, and guide public health strategies^2^ ^-^ ^8^.

The coronavirus disease 2019 (COVID-19) pandemic underscored the critical role of autopsy studies: Initially viewed as a predominantly respiratory infection, COVID-19 was identified through autopsy as a multisystemic disorder with endothelial injury, microvascular thrombosis, and widespread inflammation, informing clinical care and therapeutics^9^ ^-^ ^11^.

Conventional autopsy is the gold standard for postmortem investigation, enabling morphological, histological, biochemical, and molecular assessments; however, costly infrastructure and specialized facilities are required, particularly for highly transmissible pathogens, that are often unavailable^12^ ^-^ ^19^. For example, manipulation under biosafety level 3 conditions is required for severe acute respiratory syndrome coronavirus 2 (SARS-CoV-2), yet Brazil had no biosafety level 3 infrastructure to conduct conventional autopsies in COVID-19 cases.

Minimally invasive autopsy was developed as a safer, more acceptable alternative to conventional autopsy, and was initially used in Brazil during the yellow fever control program with a viscerotome designed for liver tissue collection; minimally invasive autopsy was subsequently expanded to multiorgan sampling with biopsy needles^20^ ^-^ ^23^. The later addition of ultrasound-guided minimally invasive tissue sampling (US-MITS) enabled improved accuracy, particularly for spleen and kidney specimens^24^.

Although US-MITS does not replace conventional autopsy, it yields samples suitable for histological, microbiological, and molecular analyses, and its use has demonstrated feasibility and safety in investigations of infectious diseases, including those for COVID-19^25^ ^-^ ^27^. The findings of recent studies have reinforced the diagnostic value of US-MITS in pulmonary pathology and in epidemic settings where complete autopsy is difficult to perform^28^ ^-^ ^30^.

Its safety, adaptability, and diagnostic value make US-MITS an important tool for epidemics involving unknown or high-risk pathogens; these characteristics support research, education, and quality control in hospitals^26^.

During the COVID-19 pandemic, a trained team implemented a straightforward US-MITS protocol for sampling of major organs and a traceable workflow from collection to slide preparation. This enabled performance metrics and iterative skill improvement to be assessed.

To encourage the formation of new teams capable of performing US-MITS, this article describes the protocol and presents data on success rates, accuracy, target tissue quantities, and common sources of contamination.

## METHODS

### Ethical considerations

This study complied with the Declaration of Helsinki and Brazilian National Health Council guidelines (Resolution 466/2012) and was approved by the Institutional Review Board of the Gonçalo Moniz Institute, FIOCRUZ-BA (CAAE: 30607920.3.0000.0040; protocol 5.325.269, 03/31/2022). Written informed consent was obtained from the next of kin prior to performing US-MITS.

### Demographic and clinical data

Between March 2021 and February 2022, US-MITS was conducted on 13 deceased inpatients with COVID-19 at the Instituto Couto Maia, Salvador, Brazil. Demographic and clinical data were retrospectively extracted from electronic records and tabulated in Microsoft Excel version 2409 spreadsheets.

### Ultrasound-guided minimally invasive tissue sampling

The inclusion criteria were that the case was one of fatal COVID-19, confirmed through RT-PCR, with consent for performing US-MITS obtained from the family of the deceased.

The procedures were performed approximately 6 to 10 hours postmortem in a 10-m² space with a hospital stretcher, instrument table, and portable ultrasound machine. Each sampling procedure lasted approximately 2 hours, and was performed by a pathologist and technician wearing full personal protection equipment. The pathologist completed 2 weeks of US-MITS training at the University of São Paulo in early 2021.

### Sample collection and processing

**FIGURE 1:**
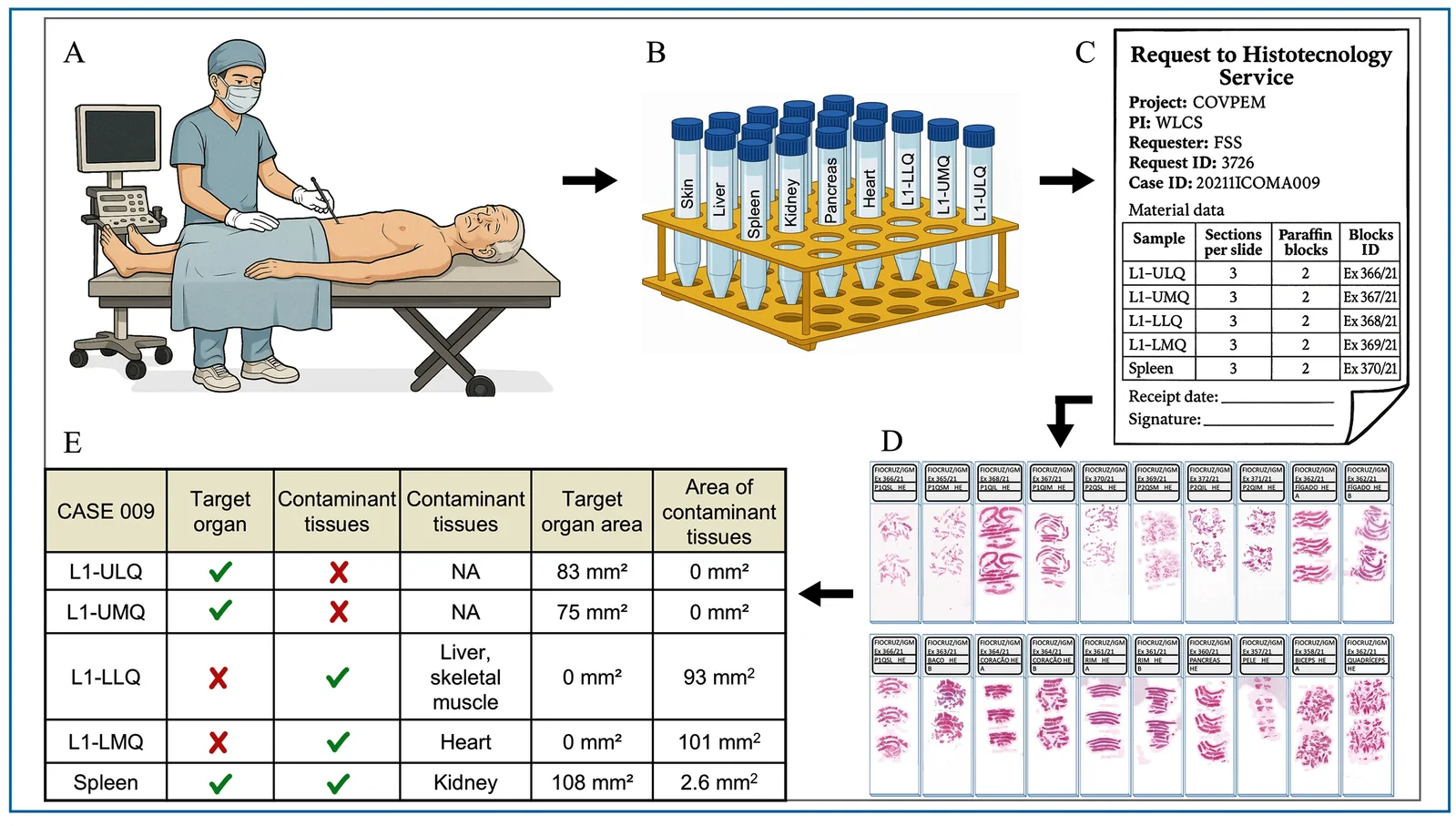
Schematic of the ultrasound-guided minimally invasive tissue sampling protocol proposed to track samples and analyses of tissue section slides. The steps shown are target organ tissue collection **(A)**, labelled tubes of target organ tissue from the attempted collection **(B)**, histological processing request form sent together with the samples collected to the histotechnology service **(C)**, tissue section slides prepared by the histotechnology service **(D)**, and record of the microscopic analysis from tissue section slides or scanned images **(E)**. **L1:** right lung; **ULQ:** upper lateral quadrant; **UMQ:** upper medial quadrant; **LLQ:** lower lateral quadrant; **LMQ:** lower medial quadrant; **NA:** not applicable.

The protocol used for sample collection and tracking is shown in Figure 1 and Supplementary Figure 1. Tissue was sampled from the skin, liver, spleen, kidneys, pancreas, lungs, heart, brain, and skeletal muscle using a 0.3-cm punch for the skin and 13G coaxial and 14G semi-automatic Tru-Cut needles for the organs. Ultrasound guidance (SonoSite M-Turbo C60x 2-5 MHz transducer, FUJIFILM Sonosite, Inc, Bothell, WA, USA) was used for sampling of the liver, spleen, kidney, and pancreas as specified. Standardized anatomical landmarks were used for four-quadrant sampling in each lung, and defined access to the heart, skeletal muscle, and brain.

Fragments were placed in 15-mL tubes with 10 mL of 4% paraformaldehyde in phosphate-buffered saline (PBS), pH 7.4 (1-2 mL of tissue per organ or quadrant, and one or two tissue fragments for the skin), and fixed for 48 to 72 hours. This was followed by routine paraffin embedding, sectioning at 2 µm, and hematoxylin and eosin staining with additional histochemical stains. Periodic acid-Schiff, Masson’s trichrome, Picrosirius Red, Heidenhain's AZAN trichrome stain reticulin, periodic Schiff-methenamine, and Sirius Red/Fast Green stains were used as required.

For electron microscopy, lung samples were processed via standard postfixation procedures in osmium tetroxide, acetone dehydration, and Poly/Bed 812 (Polysciences, Inc., Warrington, PA, USA) embedding, with semi-thin methylene blue screening and ultrathin uranyl acetate and or lead citrate staining for JEOL JEM-1230 (JEOL Ltd., Tokyo, Japan) analysis.

For SARS-CoV-2 variant calling, fragments from the right lower lateral lung (2 × 0.1 cm) were stored in RNA/DNA Shield (Zymo Research Corporation, Irvine, CA, USA), extracted using TRIzol (TRIzol is manufactured by Thermo Fisher Scientific Inc.Waltham, MA, USA)/RNeasy (QIAGEN in Hilden, Germany), amplified with Ion AmpliSeq SARS-CoV-2 Research Panel (Thermo Fisher Scientific Inc.), library-prepared with AmpliSeq Library Kit 2.0, sequenced on PGM Ion Torrent, analyzed in Torrent Suite v5.12.1 (Thermo Fisher Scientific Inc), and assigned lineages using the Phylogenetic Assignment of Named Global Outbreak Lineages tool (https://github.com/cov-lineages/pangolin).

### Histological analysis

The slides were scanned (Olympus VS100 or ZEISS AxioImager Z2) and reviewed (OlyVIA version 2.4/VSViewer, or FIJI) to delineate target and contaminant areas for measurement. Microscopic verification was performed when needed, and graphing and descriptive statistics were conducted using GraphPad Prism version 5.01 for Windows (GraphPad Software, Boston, Massachusetts, USA; www.graphpad.com).

### Success rate

The success rate for sample collection for each target organ and lung quadrant was determined as the percentage of samples collected using the following formula:

$$\frac{\;Number\;of\;cases\;in\;which\;the\;target\;organ\;or\;lung\;quadrant\;samples\;were\;collected}{Number\;of\;cases\;in\;which\;collection\;was\;attempted}\;\times 100$$

### Accuracy rate

The accuracy rate of the tissue sampling of each organ or lung quadrant was defined as the percentage of the target tissue samples relative to the total tissue sampled (target and contaminating tissues). The areas of the tissue were measured from the scanned images. To determine the target tissues, both the parenchymal and mesenchymal regions were examined. For instance, in the lung samples, alveolar areas, bronchial cartilage, and visceral pleura were considered target tissues, with contaminating tissues identified as those that did not belong to a target organ. For example, skin and skeletal muscles were considered contaminants in lung collections. The accuracy rate of the sample collection was determined using the following formula:

$$\frac{Area\;of\;target\;organ\;or\;lung\;quadrant\;tissue}{Area\;of\;target\;organ\;or\;lung\;quadrant\;tissue\;+\;area\;of\;contaminant\;tissue}\;\times \;100$$

## RESULTS

### Demographic and clinical data, and causes of death

The median age was 59 years (interquartile range [IQR], 52.5-66), 61.5% were male, and common symptoms were dyspnea (100%), cough (84.6%), and fever (61.5%). The median time from symptom onset to death was 24 days (IQR, 17.5-30.5), and immediate causes of death included acute respiratory failure (53.8%), septic shock (15.4%), hypovolemic shock (7.7%), cardiogenic shock from preexisting heart failure (7.7%), severe pancytopenia and or AIDS (7.7%), and asphyxiation resulting from airway secretions (7.7%).

### Sample collection success rate

**FIGURE 2:**
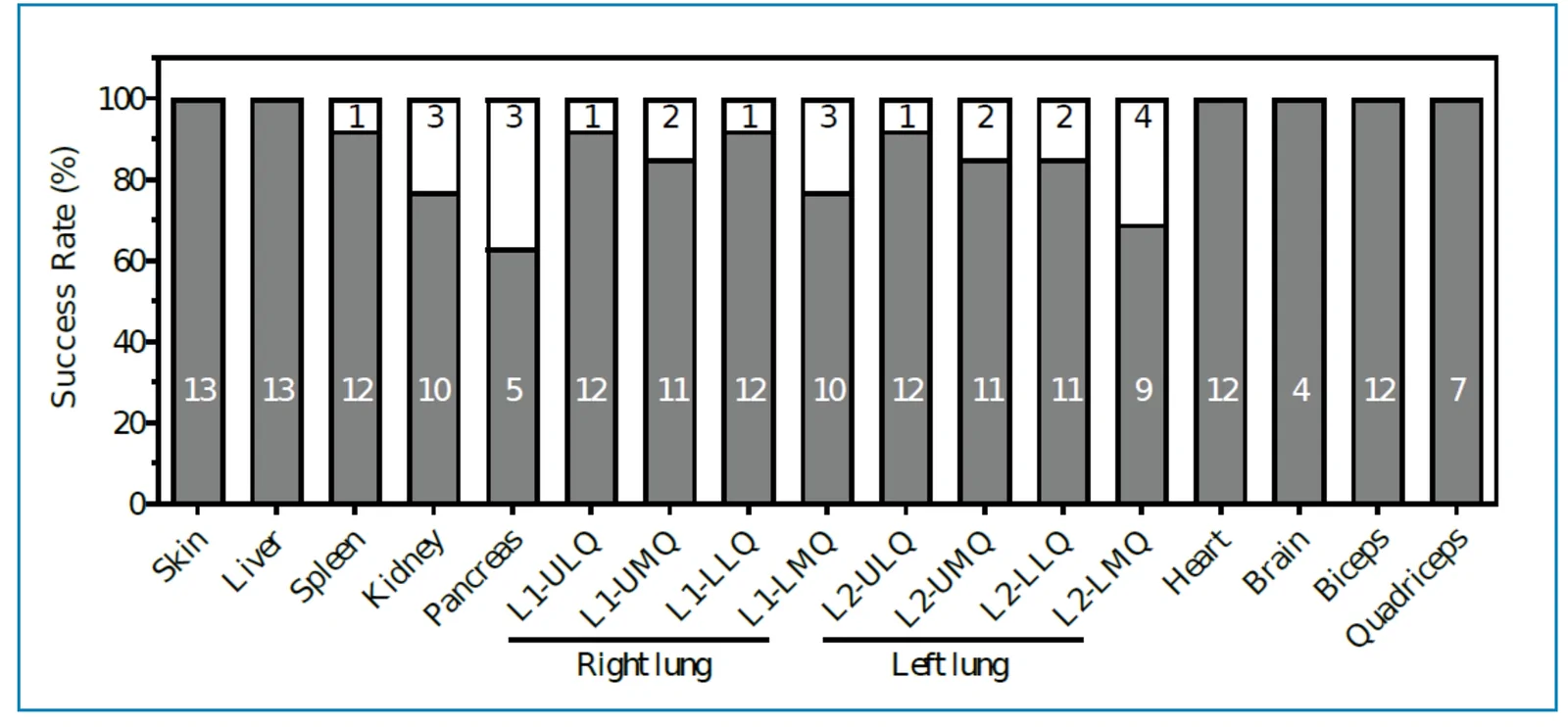
Sample collection success rate. The sample collection success rate for the attempts to collect tissue from target organs or lung quadrants is shown. The numerals inside the bars represent the number of successes (gray areas) and failures (white areas). **L1:** right lung; **L2:** left lung; **ULQ:** upper lateral quadrant; **UMQ:** upper medial quadrant; **LLQ:** lower lateral quadrant; **LMQ:** lower medial quadrant.

Success rate data for the sample collection are shown in Figure 2. Sample collection exceeded 80% for the skin (100%); liver (100%); spleen (92%) upper lateral (92%), upper medial (85%), and lower lateral (92%) quadrants of the right lung; upper lateral (92%), upper medial (85%), and lower lateral (85%) quadrants of the left lung; heart (100%); brain (100%); and skeletal muscle (100%). The success rates for the kidney (77%), pancreas (63%), lower medial quadrant of the right lung (77%), and lower medial quadrant of the left lung (69%) were all less than 80%. 

### Quantities of target tissues collected

**FIGURE 3:**
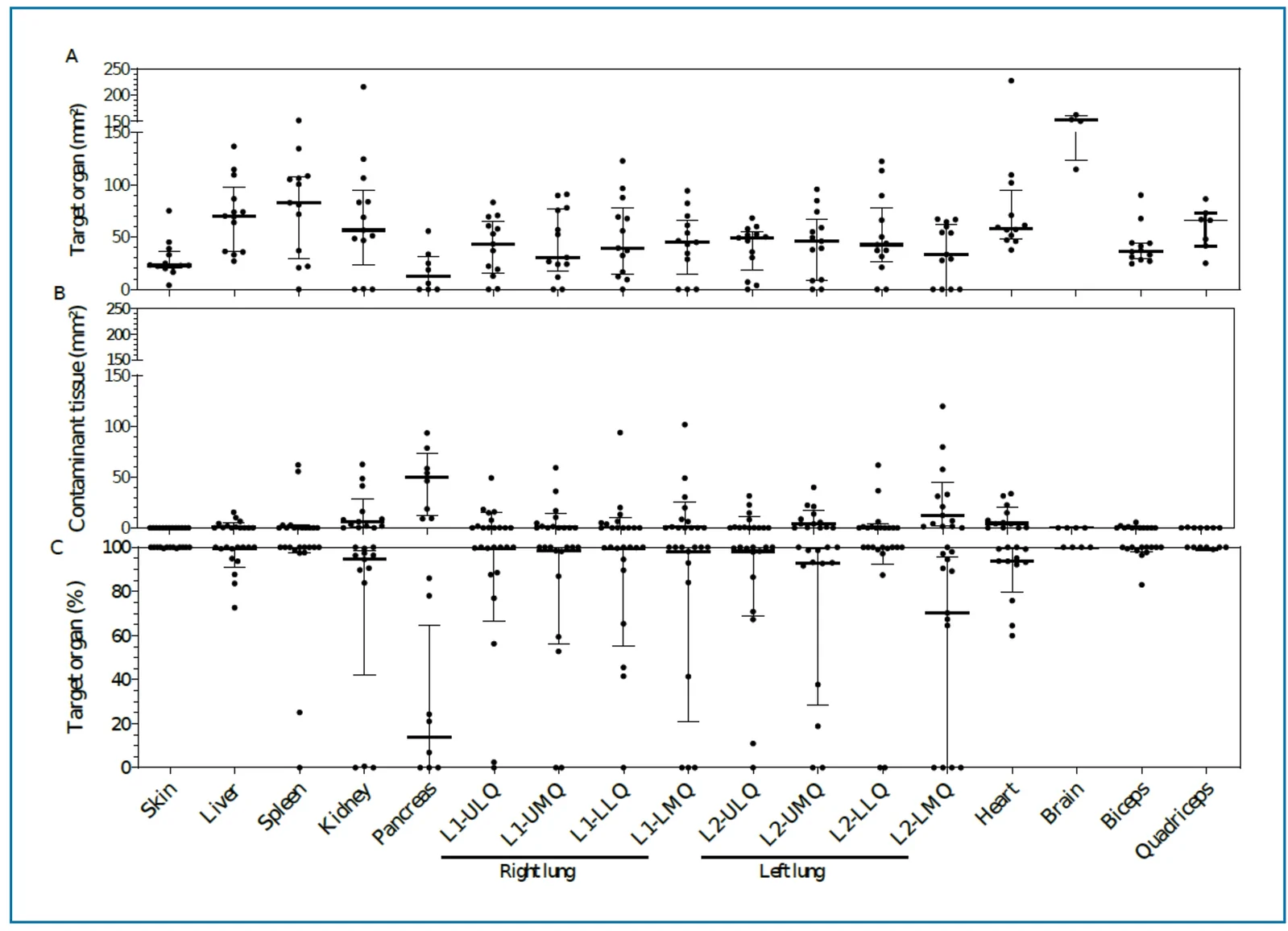
Sample collection accuracy. To evaluate the accuracy of the sample collection, the areas of the target organ **(A)** and contaminant **(B)** tissue, and percentages of target organ area in relation to contaminant tissues area **(C)** were determined. The dots, horizontal bars, and whiskers represent the values of individual cases, and medians, and 25th and 75th IQRs of the cases in which collection was attempted, respectively. **L1:** right lung; **L2:** left lung; **ULQ:** upper lateral quadrant; **UMQ:** upper medial quadrant; **LLQ:** lower lateral quadrant; **LMQ:** lower medial quadrant.

The quantities of target tissues collected are shown in Figure 3A. The target area measurements for the pancreas were the smallest (median, 12.3 mm² [IQR, 0.0-31.5]), and those for the brain were the largest (median, 152.1 mm² [IQR, 123.8-160.3]).

### Quantities and types of contaminating tissues

The quantities of contaminating tissues are shown in Figure 3B. Contaminants most frequently included the skin, skeletal muscle, adipose tissue, and fibrous connective tissue. The highest levels of contamination were observed in collections from the pancreas (≤25% of the target tissue in 6/8 cases, with 3/8 cases yielding no pancreatic tissue), often contaminated by tissue from the heart, liver, intestine, stomach, or spleen. Collections from the lower medial quadrant of the left lung were the second most contaminated samples, frequently contaminated by heart tissue (Table 1).

**TABLE 1:** Frequencies and types of contaminating tissues in attempted sample collections from different target organs.

Target organ	Contaminants											Number of US-MITS cases
	SM	AT	CT	SK	HEA	LIV	LN	INT	LUN	STO	SPL	
Skin	0	0	0	0	0	0	0	0	0	0	0	13
Liver	1	2	4	1	0	0	0	2	0	0	0	13
Spleen	3	3	2	0	1	1	1	0	1	0	0	13
Kidney	5	8	9	0	0	4	0	1	0	0	0	13
Pancreas	3	5	2	1	2	2	0	7	0	2	2	8
L1-ULQ	3	4	2	2	0	0	1	0	0	0	0	13
L1-UMQ	6	5	3	2	1	0	3	0	0	0	0	13
L1-LLQ	8	2	2	1	0	1	0	0	0	0	0	13
L1-LMQ	1	1	0	1	1	4	0	0	0	0	0	13
L2-ULQ	4	5	0	4	0	0	0	0	0	0	0	13
L2-UMQ	3	4	4	3	0	1	5	0	0	0	0	13
L2-LLQ	5	3	0	1	2	0	0	0	0	0	0	13
L2-LMQ	5	2	0	0	8	0	0	0	0	0	0	13
Heart	1	2	1	3	0	1	0	0	4	0	0	12
Brain	0	0	0	0	0	0	0	0	0	0	0	4
Biceps	0	2	1	2	0	1	0	0	0	0	0	12
Quadriceps	0	0	0	1	0	0	0	0	0	0	0	7

**L1:** right lung; **L2:** left lung; **ULQ:** upper lateral quadrant; **UMQ:** upper medial quadrant; **LLQ:** lower lateral quadrant; **LMQ:** lower medial quadrant; **SM:** skeletal muscle; **AT:** adipose tissue; **CT:** fibrous connective tissue; **SK:** skin; **HEA:** heart; **LIV:** liver; **LN:** lymph node; **INT:** intestine; **LUN:** lung; **STO:** stomach; **SPL:** spleen; **US-MITS:** ultrasound-guided minimally invasive tissue sampling. The numerals correspond to the number of cases in which contaminant tissue was detected.

### Sample collection accuracy

Collection accuracy, median-based, was categorized as less than 90%, 90% to 95%, or more than 95% (Figure 3C). The accuracies for collections from the pancreas (14%) and lower medial quadrant of the left lung (70%) were below 90%. Those for the kidney, upper medial quadrant of the left lung, and heart were between 90% and 95%; and those for the skin, liver, spleen, all quadrants of the right lung, the upper and lower lateral quadrants of the left lung, brain, and skeletal muscle were above 95%.

### Portal spaces and glomeruli

**FIGURE 4:**
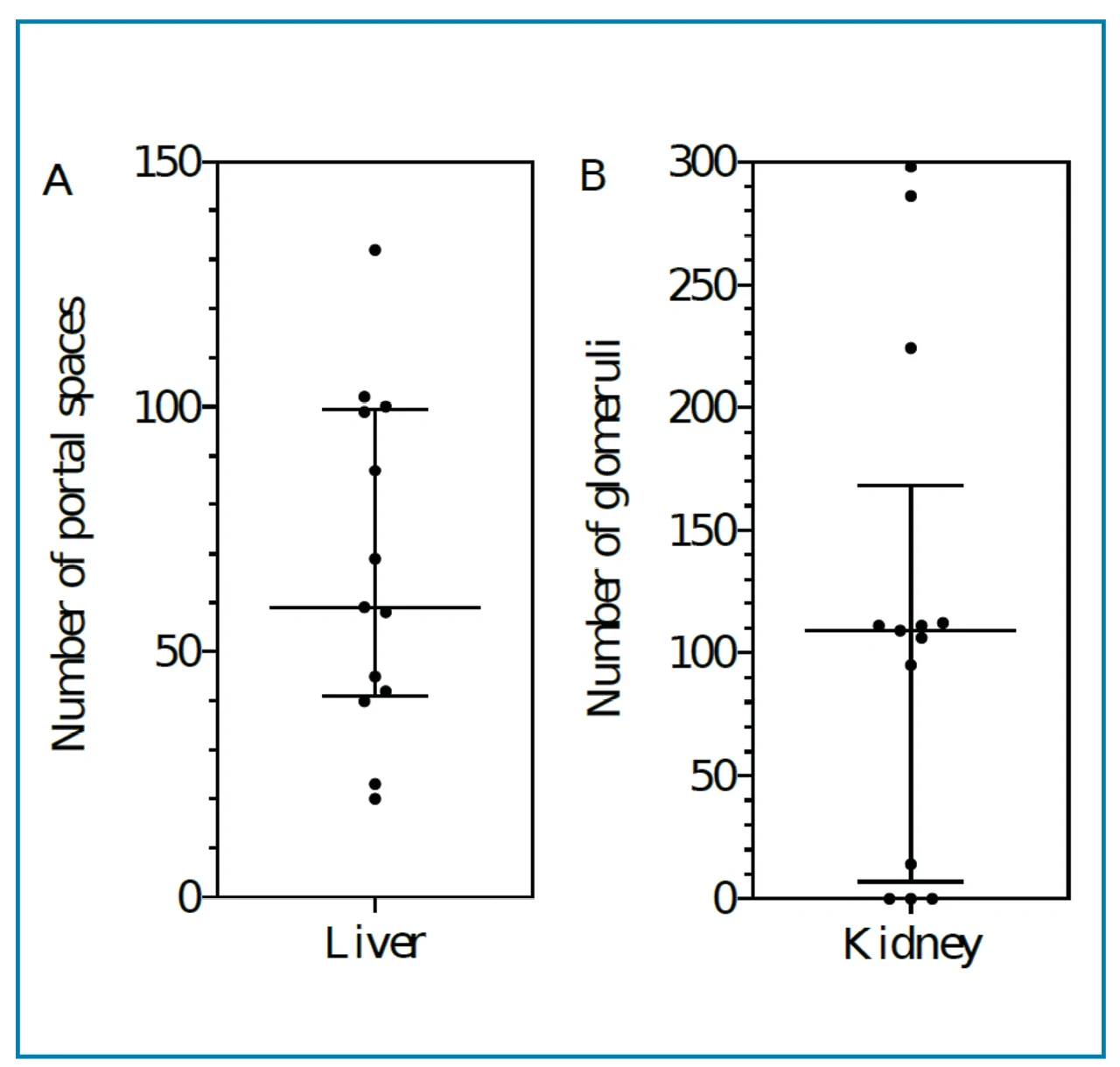
Evaluation of the adequacy of target organ sampling for pathological diagnosis. To determine if the quantity of the tissue samples collected from the liver **(A)** and kidney **(B)** was suitable for histological diagnoses, the number of portal spaces and glomeruli in the 13 cases in which ultrasound-guided minimally invasive tissue sampling was used were determined. The dots, horizontal bars, and whiskers represent the values of individual cases, and median, and 25th and 75th interquartile ranges of the cases in which collection was attempted, respectively. Adequate biopsy samples for assessment of the liver and kidney are typically defined as containing at least 11 portal spaces and 10 to 15 glomeruli, respectively.

The biopsy criteria of 11 or more portal tracts for the liver^27^ and 10 to 15 glomeruli for the kidney^31^ were adequately met across all cases. The number of portal tracts from the liver ranged from 20 to 132, and the number of glomeruli from the kidney ranged from 14 to 298 (Figure 4).

### Semi-thin section analyses

Ultrastructure sampling of the lungs (9/9 evaluable), liver (3/3 evaluable), spleen (8/9 evaluable), and kidneys (7/9 evaluable) was successful. Only isolated cases lacked target tissue on semi-thin review.

### Detection of SARS-CoV-2 in the lung

Using RT-PCR, SARS-CoV-2 was detected in all 13 cases, with specimens from one case being unsuitable for sequencing. The lineages were identified as Gamma (8/13, 61.5%), Zeta (1/13, 7.7%), and Delta (1/13, 7.7%). Lineages could not be assigned to two sequenced samples due to poor quality.

## DISCUSSION

This study presents an operationally simple, biosafe US-MITS protocol that yields multiorgan samples that are suitable for histological examination, ultrastructural analysis, and molecular detection in resource-limited tropical settings where complete autopsies are often infeasible due to biosafety, cultural, or logistical constraints^15^ ^,^ ^32^ ^,^ ^33^. The protocol incorporates sampling with a standardized tissue-tracking system to enable iterative performance improvement. These findings are consistent with those of recent studies showing that US-MITS performs well in pulmonary pathology of COVID-19 and is useful as a diagnostic strategy in outbreak and pandemic contexts^28^ ^-^ ^30^ ^,^ ^34^. Two other large minimally invasive tissue sampling studies, performed without ultrasound guidance for collection and carried out in Mozambique and in the Brazilian Amazon, also support the validity of using this approach as an alternative to conventional autopsy in resource-limited tropical settings^35^ ^,^ ^36^.

Using US-MITS can support routine postmortem surveillance during outbreaks caused by arboviruses and emerging fungal and bacterial pathogens. This facilitates minimally invasive investigations being performed to clarify cause of death, pathogen distribution, and tissue tropism^25^ ^,^ ^26^ ^,^ ^29^ ^,^ ^37^.

In the minimally invasive tissue sampling validation study conducted in the Brazilian Amazon, infectious diseases accounted for 72% of deaths. The pathogen identification rate of 87% (35/40 infectious deaths) demonstrates that this sampling method is also useful for surveilling outbreaks and characterizing regional pathogen profiles in tropical endemic settings^35^.

Across prior US-MITS studies-Fariña *et al*. (n = 100), Wong *et al*. (n = 39), Cox *et al*. (n = 95), Castillo *et al*. (n = 30), and Duarte-Neto *et al*. (n = 80)-the protocols commonly used Tru-Cut needles and ultrasound guidance for the heart, liver, spleen, and kidney; however, the objectives, operators, training, equipment, and pass numbers varied. The success rates ranged from approximately 72% to 100% (heart), 82% to 100% (lung), 97% to 100% (liver), 70% to 100% (spleen), and 67% to100% (kidney). In the 13 cases in the present study, the success rates were 100% (heart), 100% (lung), 100% (liver), 92% (spleen), and 77% (kidney), consistent with the rates previously reported^24^ ^,^ ^29^ ^,^ ^38^ ^-^ ^40^.

Targeting four bilateral anatomical lung quadrants provided comprehensive histological representation; a success rate of 80% or more was achieved in the upper and lower lateral quadrants, whereas the rates achieved in the medial lower quadrants were modestly lower due to their proximity to the liver or heart. These rates improved with operator experience in visual and or tactile cues and needle-angle adjustments.

Quantifying performance via areas calculated from digitized slides facilitated reporting of the success, accuracy, contamination, and tissue yield beyond binary adequacy. Compared with the study by Castillo *et al*., larger tissue quantities were obtained across organs by sampling up to approximately 2 mL of tissue per target region, increasing the quantity of material available for analysis at the expense of additional procedure time^40^.

Contamination-dominated by skeletal muscle, adipose, and fibrous connective tissues, and skin-was generally manageable in the histological examinations, but more consequential in the ultrastructural and transcriptomic analyses. The lowest accuracy achieved was for the pancreas (likely due to postmortem gas hindering sonographic visualization) and the lower medial quadrant of the left lung (adjacent to the heart), suggesting that gains can be made from angle optimization and path planning.

The US-MITS fragments supported transmission electron microscopy and virological testing. SARS-CoV-2 was detected in all 13 lungs, with lineage assignment possible in 10 despite the 6 to 10-hour intervals before the postmortem was conducted; this aligns with feasibility shortly after death and supports suitability for downstream molecular analyses^26^.

US-MITS is a simple, valuable technique that, with minimal training, can be successfully performed by teams. Proficiency is enhanced by using a traceable collection protocol, visual inspection, systematic recording, and iterative adjustments, enabling diagnostic sampling while minimizing contamination and reliance on full autopsies. This technique is particularly powerful for investigating infectious disease outbreaks. The use of US-MITS has also been extended to the epidemiological surveillance of arboviruses and other infectious causes of death, supporting broader implementation in death investigation services^41^.

## Data Availability

Research data is available in the body of the article.
