# Supplementary material for: Accelerating Proficiency in Minimally Invasive Autopsy for Infectious Disease Diagnosis and Research: A Strategic Framework
Source: Rev Soc Bras Med Trop. 2026 Sep 18;59:e0037-2026. doi: 10.1590/0037-8682-0037-2026 (PMC13592858; doi:10.1590/0037-8682-0037-2026)

Supplementary Figure 1. Schematic drawing of sample collection from target organs. The numbers correspond to the various target organs/lung quadrant: skin (1), liver (2), spleen (3), kidney (4), pancreas (5), upper lateral quadrant of right lung (L1-ULQ) (6), upper medial quadrant of right lung (L1-UMQ) (7), lower lateral quadrant of right lung (L1-LLQ) (8), lower medial quadrant of right lung (L1-LMQ) (9), upper lateral quadrant of left lung (L2-ULQ) (10), upper medial quadrant of left lung (L2-UMQ) (11), lower lateral quadrant of left lung (L2-LLQ) (12), lower medial quadrant of left lung (L2-LMQ) (13) heart (14), brain (15) biceps brachii (16), and quadriceps femoris (17). Source: Smart Servier (adapted).

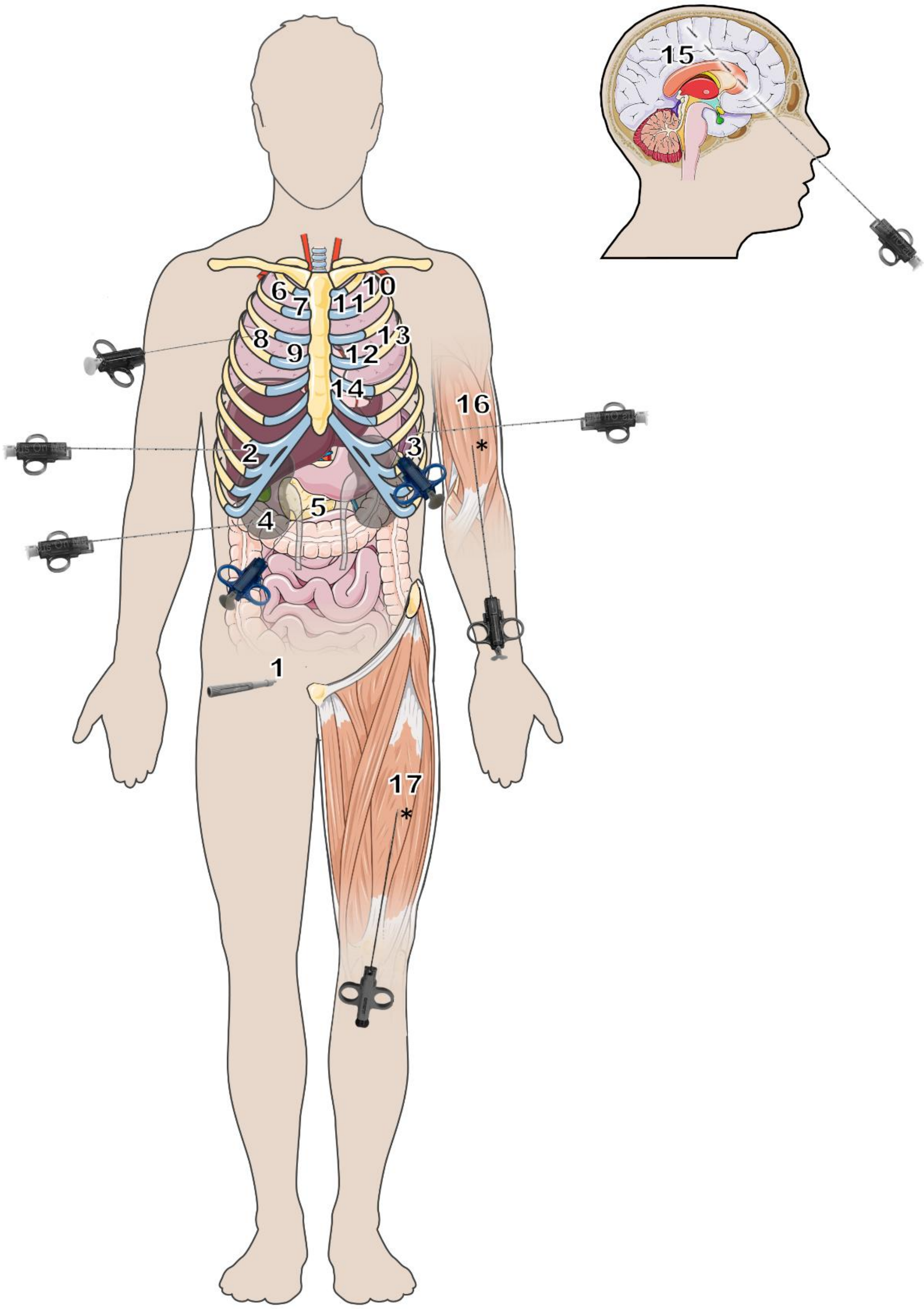

Supplement: Supplementary file 1 [file 1678-9849-rsbmt-59-e0037-2026-md1.pdf]
